# Molecular Classification of Thyroid Nodules with Indeterminate Cytology: Development and Validation of a Highly Sensitive and Specific New miRNA-Based Classifier Test Using Fine-Needle Aspiration Smear Slides

**DOI:** 10.1089/thy.2018.0254

**Published:** 2018-12-14

**Authors:** Marcos Tadeu dos Santos, Ana Lígia Buzolin, Ricardo Ribeiro Gama, Eduardo Caetano Albino da Silva, Rozany Mucha Dufloth, David Livingstone Alves Figueiredo, André Lopes Carvalho

**Affiliations:** ^1^ Department of Research and Development, Onkos Molecular Diagnostics, Ribeirão Preto/SP, Brazil.; ^2^ Department of Molecular Oncology Research Center, Barretos Cancer Hospital, Barretos/SP, Brazil.; ^3^ Department of Head and Neck Surgery, Barretos Cancer Hospital, Barretos/SP, Brazil.; ^4^ Department of Pathology, Barretos Cancer Hospital, Barretos/SP, Brazil.; ^5^ Midwestern State University (UNICENTRO), Guarapuava/PR, Brazil.

**Keywords:** miRNA, indeterminate thyroid nodule, molecular diagnostics, molecular classifier, precision endocrinology

## Abstract

***Background:*** Thyroid nodules can be identified in up to 68% of the population. Fine-needle aspiration (FNA) cytopathology classifies 20%–30% of nodules as indeterminate, and these are often referred for surgery due to the risk of malignancy. However, histological postsurgical reports indicate that up to 84% of cases are benign, highlighting a high rate of unnecessary surgeries. We sought to develop and validate a microRNA (miRNA)-based thyroid molecular classifier for precision endocrinology (mir-THYpe) with both high sensitivity and high specificity, to be performed on the FNA cytology smear slide with no additional FNA.

***Methods:*** The expression of 96 miRNA candidates from 39 benign/39 malignant thyroid samples, (indeterminate on FNA) was analyzed to develop and train the mir-THYpe algorithm. For validation, an independent set of 58 benign/37 malignant FNA smear slides (also classified as indeterminate) was used.

***Results:*** In the training set, with a 10-fold cross-validation using only 11 miRNAs, the mir-THYpe test reached 89.7% sensitivity, 92.3% specificity, 90.0% negative predictive value and 92.1% positive predictive value. In the FNA smear slide validation set, the mir-THYpe test reached 94.6% sensitivity, 81.0% specificity, 95.9% negative predictive value, and 76.1% positive predictive value. Bayes' theorem shows that the mir-THYpe test performs satisfactorily in a wide range of cancer prevalences.

***Conclusions:*** The presented data and comparison with other commercially available tests suggest that the mir-THYpe test can be considered for use in clinical practice to support a more informed clinical decision for patients with indeterminate thyroid nodules and potentially reduce the rates of unnecessary thyroid surgeries.

## Introduction

Thyroid nodules are a commonly encountered clinical problem (1,2) and the most frequent endocrine disease (3). By palpation, the prevalence ranges from 4% to 7% (4,5) and using high-resolution ultrasound nodules are found in 19%–68% of the population (6,7).

Although nodules are frequent, only 3%–7% are diagnosed as “malignant” (Bethesda class VI) when thyroid nodules are evaluated by fine-needle aspiration (FNA), with the majority of cases (approximately 60%–70%) classified as “benign” (Bethesda class II) (8). However, approximately 20%–30% of cases are indeterminate (Bethesda classes III, IV, and V) on cytology (8,9). Since the risk of malignancy (RoM) of indeterminate thyroid nodules is not low, even when considering the reclassification of NIFTP (noninvasive follicular thyroid neoplasm with papillary-like nuclear features) as benign (RoM 6%–60%) (9–12), surgery is still the current practice performed and recommended for the majority of these cases (8).

Considering that around 69% of indeterminate thyroid nodules are classified as benign by postsurgical histology, ranging from 24.8% for Bethesda class V up to 84.1% to Bethesda class III (13), many unnecessary surgeries are performed yearly. This problem is worsening: an overdiagnosis and overtreatment of thyroid nodules has been documented over the last decade as a result of a large increase in thyroid cancer screening (14), highlighting an important need to improve the diagnostic evaluations of patients with indeterminate cytological classifications on FNA in the preoperative setting.

To provide more objective information and support a more informed and personalized clinical decision, molecular tests have emerged as powerful tools to overcome this problem (15). Mutational analysis of genes such as *BRAF*, *TERT*, *RAS*, and *TP53* and several gene fusions, alone or in panels, have good specificity and positive predictive values (PPVs) and are usually used as “rule-in” tests to predict malignancy (15,16), but they are not intended to avoid unnecessary surgeries.

On the other hand, “rule-out” molecular classifier tests are good tools to help identifying benign thyroid nodules, and these tests aim to reduce unnecessary surgeries due to their high sensitivity and negative predictive values (NPVs) (17). To our knowledge, molecular classifier tests are currently only performed commercially by five centralized laboratories worldwide (18–22), which geographically and financially hinders their use by and benefit to patients from other countries. Another important limitation is that three out of the five tests require an additional biological sample, which usually means performing at least one additional FNA (18–20). Although a needle washout from the initially performed passes can be collected and stored, and can potentially avoid an additional FNA, this is not performed routinely by all centers. Currently, only two out of the five (21,23) molecular classifier tests can be performed using the cytological smear slides that were used to classify the thyroid nodule as indeterminate. These two tests are good “rule-out” options due to their high sensitivity; however, both tests are unable to achieve the proposed minimum of 80% of specificity in order to be considered a “rule-in” test option and thus perform adequately in a broad range of disease prevalence (17).

In the present study, our aim was to identify a panel of microRNAs (miRNAs) that have an distinct expression profile in benign nodules compared to malignant nodules, and to develop and validate a miRNA-based thyroid molecular classifier for a precision endocrinology (mir-THYpe) test with both high sensitivity and high specificity, which could be performed directly from the readily available cytological smear slides without the need for an additional FNA.

## Materials and Methods

### Study design and participants

A retrospective analysis was performed to identify samples from patients with thyroid nodules who were subjected to FNA procedures between January 2013 and July 2017 from which the cytopathology analysis classified the samples as “atypia of undetermined significance/follicular lesion of undetermined significance” (AUS/FLUS – Bethesda class III), “follicular or oncocytic (Hürthle cell) neoplasm/suspicious for a follicular or oncocytic (Hürthle cell) neoplasm” (FN/SFN – Bethesda class IV), or “suspicious for malignancy” (SUSP – Bethesda class V), and who had undergone total or partial thyroidectomy. Samples were obtained from the Barretos Cancer Hospital (for mir-THYpe test development and validation) and from Midwestern State University UNICENTRO (for mir-THYpe test validation).

Samples were tested at Barretos Cancer Hospital, in a laboratory certified according to the provisions of the College of American Pathologists, United Kingdom National External Quality Assessment Service, and the European Molecular Genetics Quality Network. The study was approved by the board of the investigational ethics committee and listed under CAAE 52739416.5.0000.5437. All patients provided written informed consent to participate in the study.

### Sample selection and evaluation

To confirm whether the nodule described in the surgical pathology report of the removed tissue corresponded to the same nodule biopsied in the FNA, all eligible cases were reviewed. The nodule description obtained by ultrasonography was also compared with the FNA cytology analysis report and the final postsurgical report. The sample was included in the study only if the FNA cytology report and other characteristics, such as size and localization, corresponded to the nodule described in the final surgical pathology report. Samples in which the final diagnosis did not correspond to the FNA biopsy, as well as those samples in which it was impossible to define with absolute certainty that the two samples were matching, due to more than one nodule in the same patient or to a lack of details in the description of the reports, were excluded.

All selected samples had their respective FNA cytology slides reviewed by a second independent pathologist. The reviews were double-blinded, without the reviewing pathologist being aware of the original classification. Samples whose review reports were concordant with the original were immediately included in the study. Samples with discordance with the original report were subjected to blinded review by an independent third pathologist in order to make a decision. If the opinion from the third pathologist was concordant with any of the two previous opinions, the sample was included in the study with its classification. If discordant with both previous opinions, the sample was excluded from the study. To confirm the postsurgical pathology reports, the histological slides of the nodules corresponding to the FNA smears were also reviewed according to the same approach.

For the development and training phase of the mir-THYpe test (training set), only cryopreserved and FFPE (formalin-fixed paraffin-embedded) tissues were used. For the validation phase of the mir-THYpe test (validation set), only FNA cytology smear slides were used, and they were used only when at least two representative slides were available: one was to be used in the study, and the other was to be kept in the patient records. None of the samples used in the validation set were used for algorithm development or training. Demographic patient characteristics and clinical data are shown in Table 1.

**Table 1.** Demographic and Clinical Characteristics of the Study Cohorts

	*Total*		*Training set (post-surgery tissue)*		*Validation set (FNA smear slides)*	
*Variable*	*Number*	*%*	*Number*	*%*	*Number*	*%*
**Cohort**						
Samples	173	–	78	–	95	–
Patients	163	–	78	–	85	–
**Sex**						
Male	27	16.6%	13	16.7%	14	16.5%
Female	136	83.4%	65	83.3%	71	83.5%
**Age (years)**						
<20	6	3.7%	2	2.6%	4	4.7%
20-54	87	53.4%	40	51.3%	47	55.3%
≥ 55	70	42.9%	36	46.2%	34	40.0%
**Nodule size (cm)**^a^						
<2	88	50.9%	42	53.8%	46	48.4%
2-4	64	37.0%	29	37.2%	35	36.8%
>4	20	11.6%	7	9.0%	13	13.7%
**TNM staging**^b^						
T1	45	59.2%	20	51.3%	25	67.6%
T2	23	30.3%	15	38.5%	8	21.6%
T3	7	9.2%	3	7.7%	4	10.8%
T4	1	1.3%	1	2.6%	0	0.0%
N0	68	89.5%	38	97.4%	30	81.1%
N1	8	10.5%	1	2.6%	7	18.9%
M0	74	97.4%	38	97.4%	36	97.3%
M1	2	2.6%	1	2.6%	1	2.7%
**Stage grouping**^b^						
I	71	93.4%	37	94.9%	34	91.9%
II	4	5.3%	2	5.1%	2	5.4%
IVb	1	1.3%	0	0.0%	1	2.7%

^a^ We could not retrieve the nodule size of one sample (Bethesda class IV, benign, colloid goiter) from the validation set.

^b^ TNM Staging and Stage Grouping refers only to malignant samples (training set, 39; validation set, 37).

FNA, fine needle aspiration.

### RNA extraction

Prior to extraction, all cases were evaluated by a pathologist to analyze the percentage of tumor tissue present in the sample. All samples used had more than 70% of tumor tissue. Total RNA from the FFPE samples was isolated from two to six 10-μm tissue sections using the RecoverAll Total Nucleic Acid Isolation Kit for FFPE (Ambion, Carlsbad, CA). Total RNA extraction from cryopreserved samples was performed using the QIAsymphony automated system (QIAGEN, Hilden, Germany), using the microRNA enrichment extraction protocol. Total RNA from the FNA cytology smear slides (stained by Papanicolaou, Giemsa, or Diff-Quik methods) was performed with the TRIzol reagent (Thermo Scientific, Waltham, MA) protocol. The cells were scraped only from marked zones delimited by a cytopathologist, with a minimum of at least six groups of 10 thyroid cells being acceptable to perform the protocol. Quantification of the total RNA extracted was performed using the Qubit 2.0 equipment (Thermo Scientific, Waltham, MA). RNA was resuspended to a final volume of 30 μL of ultrapure D/RNase-free water. The entire process was carried out according to the techniques established in the RNA manipulation protocols, in an exclusive laboratory environment, and with the aid of tubes, pipettes, tips, and exclusive supports that had been previously sanitized with the RNAseZAP product (Ambion, Carlsbad, CA). All steps were performed according to the manufacturer's instructions.

### Reverse transcription cDNA synthesis and preamplification

Twenty nanograms of total RNA from each sample were used for cDNA synthesis. The reaction used a pool of specific reverse transcription (RT) predesigned inventoried primers (Thermo Scientific, Waltham, MA) to select only the miRNAs of interest (see the list of targets in Supplementary Table S1; Supplementary Data are available online at www.liebertpub.com/thy), and the reactions were performed using the TaqMan microRNA Reverse Transcription Kit (Thermo Scientific, Waltham, MA). Subsequently, the RT-PCR products were subjected to the preamplification stage, with a pool of specific predesigned TaqMan inventoried assays (Thermo Scientific, Waltham, MA) and the Pre-Amp Master Mix 2 × (Applied Biosystems, Carlsbad, CA). All reactions were performed according to the manufacturer's instructions.

### Real-time PCR

For the training set, customized TaqMan Low-Density Array (TLDA) 384-well microfluidic cards with inventoried predesigned assays were designed using the Custom TaqMan Gene Expression Array Card service from Life Technologies, Carlsbad, CA (format 96a). For the validation set, 96-well fast plates were used to carry out the reactions using specific predesigned TaqMan inventoried individual assays (Thermo Scientific, Waltham, MA). The preamplified cDNAs were mixed with 2 × TaqMan Gene Expression Master Mix (Applied Biosystems, Carlsbad, CA), and TLDA cards or 96-well plates analyzed the QuantStudio 12K Real-Time PCR System (Applied Biosystems, Carlsbad, CA). Samples were heated at 50°C for 2 minutes and 95°C for 10 minutes and then subjected to 50 cycles of 95°C for 15 seconds and 60°C for 1 minute. The Ct (cycle threshold) of each gene was obtained by setting the fixed threshold at 0.05.

### miRNA target selection

For the training set, we selected an exploratory panel of 96 miRNA candidates to be analyzed on the TLDA cards. For this first panel, we used an ensemble of two different approaches. First, we used the FirePlex Discovery Engine (www.fireflybio.com) to filter miRNAs that were frequently cited in the literature using “thyroid nodules,” “thyroid cancer,” thyroid biomarker,” and “thyroid miRNA” as keywords. Second, we performed a comprehensive search of PubMed (www.ncbi.nlm.nih.gov/pubmed), looking not only for the frequencies of the miRNAs cited in relevant studies but also for the targets that were described to be differentially expressed in benign and malignant nodules or as good candidates for normalizers (housekeeping targets).

All targets that were not amplified in at least 95% of the cryopreserved and FFPE samples used for the training set were excluded and were not even considered as candidates to constitute the mir-THYpe algorithm. The top 10 targets with lower Ct standard deviations across all benign and malignant samples were assigned as normalizer candidates. All remaining miRNAs were assigned to be used as discriminator candidates.

### miRNA-based thyroid molecular classifier algorithm development and training

For each sample from the training set (FFPE and cryopreserved), we produced a series of features by normalizing the Ct value of each discriminator candidate to all possible combinations of the average of Ct values from the 10 normalizer candidates expressed as an exponential delta cycle threshold (ΔCt) = 2^^(Ct average normalizers – Ct discriminator)^. The best features were selected based on the mutual information filter-based method (24) and classifiers were generated using the Tree-based algorithms (25). The algorithm tree with better clinical performance, based on the 10-fold cross-validation analysis, was locked and then challenged with the normalized data obtained from the samples from the validation set cohort (FNA cytology smear slides). The classifier algorithm tree performance was evaluated per the NPV, PPV, sensitivity, specificity, negative and positive likelihood ratios, accuracy, and area under the curve. Supplementary Figure S1 shows an unsupervised hierarchical clustering analysis of normalized expression data from discriminator miRNAs used on the mir-THYpe algorithm.

### Statistical analyses

Statistical analyses were performed using the R software, an open-source statistical programming environment. Confidence intervals for sensitivity, specificity, and accuracy were “exact” Clopper-Pearson confidence intervals. Confidence intervals for the likelihood ratios were calculated using the “Log method” (26). Confidence intervals for the predictive values were the standard logit confidence intervals (27). Bayes' theorem analysis was based on Hall (28).

## Results

From the 1205 patients identified with both thyroid FNA cytology and postsurgical histology results available during the period of interest, 272 samples (22.5%) were classified as AUS/FLUS (Bethesda class III), FN/SFN (Bethesda class IV), or SUSP (Bethesda class V). Of these samples, 60 were excluded due to a lack of at least two representative FNA smear slides or the presence of multinodular thyroid glands for which the description of the punctured FNA nodule was not sufficient to correlate it with the available FFPE or cryopreserved tissue. Another 20 samples were excluded during the pathology review process, in which the final consensus was either “benign” (Bethesda class II) or “malignant” (Bethesda class VI), and the other 19 samples were excluded due to the low quality of the RNA extraction and/or real-time PCR amplification issues.

The remaining 173 samples were split into two cohorts. The first cohort, used to develop and train the mir-THYpe algorithm (training set), comprised 78 samples (from 78 patients), with 39 benign and 39 malignant samples (50% cancer prevalence); 50% of the samples were from FFPE, and 50% were from cryopreservation. The second cohort, used to validate the mir-THYpe algorithm (validation set), comprised 95 samples (from 85 patients), with 58 benign and 37 malignant samples (38.9% cancer prevalence), all from the FNA cytology smear slides stored at the Pathology Department. The Supplementary Table S2, shows the details of the histological subtypes and Bethesda class compositions of each cohort.

### miRNA target selection, feature generation, and mir-THYpe development

From the first exploratory list of 96 miRNA candidates (Supplementary Table S1), 31 miRNAs were excluded since no amplification was observed on at least 95% of the 78 cryopreserved and FFPE samples (numbers 66–96 in Supplementary Table S1). From the remaining 65 miRNA candidates, 10 were selected as normalizer candidates (numbers 56–65) based on the standard deviations, and the other 55 miRNAs (numbers 1–55) were used as discriminator candidates.

The Cts from the 55 discriminator miRNAs, when normalized to all of the possible combinations of the average Ct values from the 10 normalizer miRNAs, generated 9625 candidate features. The tree-based model created with the better clinical performance utilized only five features comprising 11 targets, with 6 used as normalizers (let-7a, miR-103, miR-125a-5p, let-7b, miR-145, and RNU48) and 5 used as discriminators (miR-146b, miR-152, miR-155, miR-200b, and miR-181b).

### mir-THYpe algorithm performance

#### Training set

Table 2 summarizes all of the statistical parameters considered in the analysis of the clinical performance of the mir-THYpe algorithm. Table 3 summarizes the performance of the mir-THYpe algorithm in each cohort and for each histological subtype. When evaluated with the 10-fold cross-validation method using the training set data, the mir-THYpe algorithm was able to correctly classify 71/78 samples (91.0% accuracy [95% confidence interval (CI) 82.4–96.3]). Of the 39 malignant samples, 35 were correctly classified, yielding a sensitivity of 89.7% [95% CI 75.8–97.1]. Of the 39 benign samples, 36 were correctly classified, yielding a specificity of 92.3% [95% CI 79.1–98.4]. Considering the cancer prevalence distribution of the training set cohort (50.0% [95% CI 38.5–61.5]), the mir-THYpe algorithm also showed a high NPV (90.0% [95% CI 78.0–95.8]) and PPV (92.1% [95% CI 79.6–97.2]). Twenty out of the 22 AUS/FLUS (Bethesda class III) samples were correctly classified (90.9% [95% CI 70.8–98.9]) as well as 28 out of the 32 FN/SFN (Bethesda class IV) and 23 out of the 24 SUSP (Bethesda class V) samples were also correctly classified (87.5% [95% CI 71.0–96.5] and 95.8% [95% CI 78.9–99.9] respectively).

**Table 2.** Statistical Performance of mir-THYpe

	*Training set (postsurgical tissue)*		*Validation set (FNA smear slides)*	
*Statistical parameter*	*Value*	*[95% CI]*	*Value*	*[95% CI]*
Sensitivity	89.7%	[75.8–97.1]	94.6%	[81.8–99.3]
Specificity	92.3%	[79.1–98.4]	81.0%	[68.6–90.1]
NPV	90.0%	[78.0–95.8]	95.9%	[85.9–98.9]
PPV	92.1%	[79.6–97.2]	76.1%	[65.0–84.5]
Negative likelihood ratio	0.11	[0.04–0.28]	0.07	[0.02–0.26]
Positive likelihood ratio	11.67	[3.91–34.78]	4.99	[2.91–8.54]
Accuracy	91.0%	[82.4–96.3]	86.3%	[77.7–92.5]
Area under the curve	0.91	[0.82–0.96]	0.88	[0.79–0.94]
Cancer Prevalence	50.0%	[38.5–61.5]	38.9%	[29.1–49.5]

AUC, area under the curve; NPV, negative predictive value; PPV, positive predictive value.

**Table 3.** The mir-THYpe Accuracy for Each Thyroid Histological Subtype and Bethesda Classes

	*Training set (postsurgical tissue)*								*Validation set (FNA smear slides)*							
	*Samples correct classified / total number of samples (%)*								*Samples correct classified / total number of samples (%)*							
*Histological subtypes*	*AUS/FLUS (III)*		*FN/SFN (IV)*		*SUSP (V)*		*Total*		*AUS/FLUS (III)*		*FN/SFN (IV)*		*SUSP (V)*		*Total*	
**Benign**	**14/14**	**(100%)**	**15/18**	**(83.3%)**	**07/07**	**(100%)**	**36/39**	**(92.3%)**	**14/17**	**(82.4%)**	**31/39**	**(79.5%)**	**2/2**	**(100%)**	**47/58**	**(81.0%)**
Hüthle cell adenoma	1/1	(100%)	3/3	(100%)	0/0	n.a.	4/4	(100%)	0/1	(0%)	7/7	(100%)	0/0	n.a.	7/8	(87.5%)
Follicular adenoma	3/3	(100%)	1/1	(100%)	0/0	n.a.	4/4	(100%)	2/2	(100%)	7/12	(58.3%)	0/0	n.a.	9/14	(64.3%)
Colloid goiter	5/5	(100%)	3/6	(50.0%)	4/4	(100%)	12/15	(80.0%)	3/4	(75.0%)	5/6	(83.3%)	2/2	(100%)	10/12	(83.3%)
Adenomatous goiter/ follicular hyperplasia	1/1	(100%)	6/6	(100%)	1/1	(100%)	8/8	(100%)	7/8	(87.5%)	6/6	(100%)	0/0	n.a.	13/14	(92.9%)
Hashimoto's thyroiditis	1/1	(100%)	0/0	n.a.	0/0	n.a.	1/1	(100%)	0/0	n.a.	1/1	(100%)	0/0	n.a.	1/1	(100%)
Lymphocytic thyroiditis	2/2	(100%)	1/1	(100%)	2/2	(100%)	5/5	(100%)	2/2	(100%)	5/7	(71.4%)	0/0	n.a.	7/9	(77.8%)
Chronic thyroiditis	1/1	(100%)	1/1	(100%)	0/0	n.a.	2/2	(100%)	0/0	n.a.	0/0	n.a.	0/0	n.a.	0/0	n.a.
**Malignant**	**6/8**	**(75.0%)**	**13/14**	**(92.9%)**	**16/17**	**(94.1%)**	**35/39**	**(89.7%)**	**1/1**	**(100%)**	**5/6**	**(83.3%)**	**29/30**	**(96.7%)**	**35/37**	**(94.6%)**
Papillary thyroid carcinoma, usual type	3/3	(100%)	2/2	(100%)	11/11	(100%)	16/16	(100%)	1/1	(100%)	0/0	n.a.	18/18	(100%)	19/19	(100%)
Papillary thyroid carcinoma, follicular variant	3/3	(100%)	6/6	(100%)	5/5	(100%)	14/14	(100%)	0/0	n.a.	1/1	(100%)	11/11	(100%)	12/12	(100%)
Follicular thyroid carcinoma, widely invasive	0	n.a.	2/2	(100%)	0/0	n.a.	2/2	(100%)	0/0	n.a.	0/0	n.a.	0/0	n.a.	0/0	n.a.
Follicular thyroid carcinoma, microinvasive	0	n.a.	0/0	n.a.	0/0	n.a.	0/0	n.a.	0/0	n.a.	1/1	(100%)	0/0	n.a.	1/1	(100%)
Follicular thyroid carcinoma, oncocytic variant	0	n.a.	3/4	(75.0%)	0/1	(0%)	3/5	(60.0%)	0/0	n.a.	1/1	(100%)	0/0	n.a.	1/1	(100%)
NIFTP	0	n.a.	0/0	n.a.	0/0	n.a.	0/0	n.a.	0/0	n.a.	2/2	(100%)	0/1	(0%)	2/3	(66.7%)
Medullary thyroid carcinoma	0/2	(0%)	0/0	n.a.	0/0	n.a.	0/2	(0%)	0/0	n.a.	0/0	n.a.	0/0	n.a.	0/0	n.a.
Insular thyroid carcinoma	0	n.a.	0/0	n.a.	0/0	n.a.	0/0	n.a.	0/0	n.a.	0/1	(0%)	0/0	n.a.	0/1	(0%)
**Total**	**20/22**	**(90.9%)**	**28/32**	**(87.5%)**	**23/24**	**(95.8%)**	**71/78**	**(91.0%)**	**15/18**	**(83.3%)**	**36/45**	**(80.0%)**	**31/32**	**(96.9%)**	**82/95**	**(86.3%)**

AUS/FLUS, atypia of undetermined significance/follicular lesion of undetermined significance; FN/SFN, follicular or oncocytic (Hürthle cell) neoplasm/suspicious for a follicular or oncocytic (Hürthle cell) neoplasm; n.a., not available; NIFTP, noninvasive follicular thyroid neoplasm with papillary-like nuclear features; SUSP, suspicious for malignancy.

#### Validation set

After development and training, the mir-THYpe algorithm was locked, and the clinical performance analysis was performed by challenging it with totally new data obtained from the validation set FNA cytology smear slides. Here, the mir-THYpe algorithm was able to correctly classify 82/95 samples (86.3% accuracy [95% CI 77.7–92.5]). From the 37 malignant samples, 35 were correctly classified, yielding a sensitivity of 94.6% [95% CI 81.8–99.3]. The only two malignant samples incorrectly classified as benign (false negative) comprised one NIFTP (which was recently reclassified as a premalignant lesion, which requires surgery and a histological diagnosis) and one insular carcinoma case. Of the 58 benign samples, 47 were correctly classified, yielding a specificity of 81.0% [95% CI 68.6–90.1]. The 11 benign samples (from 9 patients) incorrectly classified as malignant (false positive [FP]) included four follicular adenomas, three cases of lymphocytic thyroiditis, two colloid goiters, one Hürtle cell adenoma, and one adenomatous goiter. Considering the cancer prevalence distribution of the validation set cohort (38.9% [95% CI 29.1–49.5]), the mir-THYpe algorithm also showed a high NPV (95.9% [95% CI 85.9–98.9]) and PPV (76.1% [95% CI 65.0–84.5]). Fifteen out of the 18 AUS/FLUS (Bethesda class III) samples were correctly classified (83.3% [95% CI 58.6–96.4]), and 36 out of the 45 FN/SFN (Bethesda class IV) and 31 out of the 32 SUSP (Bethesda class V) samples were also correctly classified (80.0% [95% CI 65.4–90.4] and 96.9% [95% CI 83.8–99.9] respectively).

#### Comparison with other molecular classifiers

**FIG. 1.**
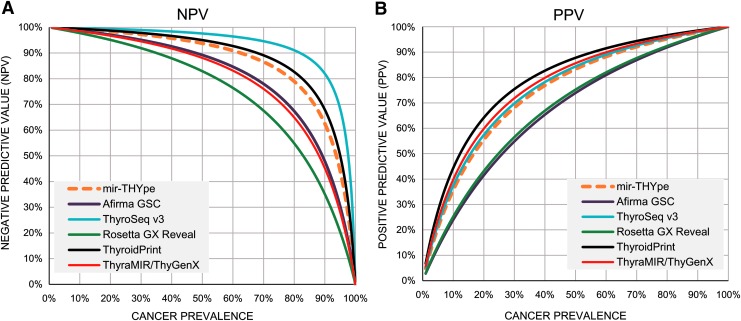
Bayes' theorem analysis showing the theoretical performance between the mir-THYpe and the other commercially available molecular classifier tests for all possible cancer prevalences. (**A**) Estimated negative predictive values (NPV). (**B**) Estimated positive predictive value (PPV). For the Rosetta GX Reveal test, we consider the entire validation set (*n* = 189).

We compiled the latest data published and compared the statistical parameters reached by the FNA validation set cohorts from mir-THYpe and another five molecular classifiers (Table 4). We observed that the cancer prevalence from each study varied from 23.7% (Afirma GSC) to 52.6% (ThyroSeq v3), which made evaluating and statistically comparing the clinical performance of each test difficult. Using Bayes' theorem, we predicted the NPV (Table 5 and Fig. 1A) and the PPV (Fig. 1B) of each test for different cancer prevalence rates to enable a fair statistical comparison. At the cancer prevalence used by the Afirma GSC study (23.7%), all tests were predicted to perform with an NPV greater than 90%. However, when considering the cancer prevalence used by the ThyroSeq v3 study (52.6%), only the mir-THYpe, ThyroSeq v3, and ThyroidPrint tests were predicted to perform with an NPV greater than 90%.

**Table 4.** Comparison of the Main Characteristics Between the mir-THYpe and the Other Commercially Available Molecular Classifier Tests

*Performance in the study FNA validation set cohort*	*Molecular classifiers*					
	*mir-THYpe*	*ThyroSeq v3^a^(19)*	*ThyroidPrint^b^ (20)*	*ThyraMIR / ThyGenX^c^ (21)*	*Rosetta GX Reveal^d,e^ (22)*	*Afirma GSC^f^ (18)*
“Rule-in” test?^§^	Yes	Yes	Yes	Yes	No	No
“Rule-out” test?^§^	Yes	Yes	Yes	No	No	No
Performed from FNA smear slides?	Yes	No	No	Yes (23)	Yes	No
Sensitivity	94.6%	98.0%	95.5%	88.6%	85.2%	91.1%
Specificity	81.0%	81.8%	86.7%	85.1%	71.9%	68.3%
NPV	95.9%	97.4%^*^	97.5%	94.0%	91.1%	96.1%
PPV	76.1%	85.7%^*^	77.8%	73.8%	59.1%	47.1%
Cancer prevalence	38.9%	52.6%	32.8%	32.1%	32.3%	23.7%
Number of samples in the study	95	175	67	109	189	190
Out-of-network cost (29)^†^	$	$$$	n.a.	$$$^**^	$$	$$$$

^a^ ThyroSeq (CBLPath, Inc., Rye Brook, NY, and University of Pittsburgh Medical Center, Pittsburgh, PA).

^b^ ThyroidPrint (GeneproDx, Inc, Santiago, Chile).

^c^ ThyraMIR/ThyGenX (Interpace Diagnostics, Inc, Parsippany, New Jersey).

^d^ Rosetta GX Reveal (Rosetta Genomics, Inc, Philadelphia, Pennsylvania).

^e^ Considering the entire validation set (*n* = 189).

^f^ Afirma (Veracyte, Inc, South San Francisco, California).

^§^ According to the thresholds proposed by Vargas-Salas and colleagues (17) in 2018.

^*^ Calculated based on Bayes' theorem, using the cancer prevalence, sensitivity, and specificity published in Nikiforova *et al.* (19).

^**^ Aggregated price of ThyraMIR ($$$) + ThyGenX ($).

^†^ The prices are typically different from payers' reimbursement schedules. Price ranges in US dollars: $, 0–1999; $$, 2000–3999; $$$, 4000–5999; $$$$, >6000 (29).

**Table 5.** Comparison of the Theoretical NPV Performance Between mir-THYpe and the Other Commercially Available Molecular Classifier Tests

	*Cancer prevalence in FNA validation set cohort*	*38.9% [mir-THYpe]*	*52.6% [ThyroSeq v3 (19)]*	*32.8% [ThyroidPrint (20)]*	*32.1% [ThyraMIR/ ThyGenX (21)]*	*32.3% [Rosetta GX Reveal^a^ (22)]*	*23.7% [Afirma GSC (18)]*
Theoretical NPV performance (Bayes' theorem)	mir-THYpe	**95.9%**	93.1%	96.8%	96.9%	96.9%	98.0%
	ThyroSeq v3	98.5%	**97.4%**^b^	98.8%	98.9%	98.8%	99.2%
	ThyroidPrint	96.8%	94.5%	**97.5%**	97.6%	97.6%	98.4%
	ThyraMIR / ThyGenX	92.1%	87.0%	93.8%	**94.0%**	94.0%	96.0%
	Rosetta GX Reveal^a^	88.4%	81.4%	90.9%	91.1%	**91.1%**	94.0%
	Afirma GSC	92.3%	87.4%	94.0%	94.2%	94.1%	**96.1%**

The theoretical NPV was calculated based on Bayes' theorem using the sensitivity, specificity, and cancer prevalence in the FNA validation set cohort of each study. Values highlighted in bold correspond to the observed NPV values on the specific cancer prevalence from the respective study.

^a^ Considering the entire validation set (*n* = 189).

^b^ Calculated based on Bayes' theorem, using the cancer prevalence, sensitivity, and specificity published in Nikiforova *et al.* (19).

## Discussion

Here, we present the results of the development and validation of a new molecular classifier test in precision endocrinology for indeterminate thyroid nodules (mir-THYpe) that analyzes the expression profiles of 11 miRNAs obtained from the same FNA cytology smear slides used to classify the thyroid nodule as indeterminate. This approach has the advantage that there is no need for a repeat FNA, which may be required with other methods if samples are not collected prospectively. In order to focus on available cytology slides, our mir-THYpe test was developed to scrape only thyroid cells in marked zones delimited by a cytopathologist.

Since this is a retrospective study, we decided to apply very rigid sample inclusion criteria to guarantee that (1) the available FNA smear slide was truly correlated with the postsurgical nodule that was biobanked, (2) the assigned Bethesda description/class and histological subtyping classification were accurate enough to be used as a gold standard, and (3) the RNA extraction and/or real-time PCR amplifications had enough quality. To meet these criteria, 99 out of the 272 indeterminate samples initially identified (36.4%) were excluded, limiting the number of AUS/FLUS (Bethesda class III) malignant nodules in both cohorts. Although limited, six out of the eight samples in the training set and a single sample in the validation set were correctly classified as malignant. This availability limitation was also observed for SUSP (Bethesda class V) benign nodules, but 100% of the seven samples in the training set and the two samples in the validation set were correctly classified as benign.

The distribution of the number of malignant versus benign samples (cancer prevalence) in the training set was different from the distribution in the validations set. The analysis of the first cohort (50.0% cancer prevalence) was aimed to provide the same amount of information from both classes to allow similar learning for the algorithm training, which was confirmed when the 10-fold cross-validation test achieved high and similar performance values in term of the sensitivity (89.7%) and specificity (92.3%). The analysis of the second cohort (38.9% cancer prevalence) was aimed to have a similar distribution observed to that in the Barretos Cancer Hospital (approximately 44% cancer prevalence) and to be inside the 20%–40% cancer prevalence, which is the range for indeterminate nodules reported in most clinical centers (20). The observed clinical performance of the validation set cohort, in which both the sensitivity (94.6%) and specificity (81.0%) were high and close to the values observed in the training set, is good evidence that the data from both cohorts are consistent. Although the data for the training set was generated only with postsurgical tissues and although the data for the validation set was generated only by FNA smear slides, the key to reaching this level of concordance between both cohorts was the use of good-quality normalization data, and this was only possible by using, among our 11-miRNA panel, more normalizer biomarkers (6) than discriminator biomarkers (5).

Considering that the specificity is the ratio between the FP and the sum of true negatives (TN) with the FP (specificity = FP/[FP+TN]), and that virtually all FP patients will undergo thyroidectomy surgery, the sensitivity is also a parameter to estimate the potential number of unnecessary surgeries that can be avoided if the test is performed for 100% of the patients with indeterminate thyroid nodules. In this situation, the mir-THYpe test has potential to reduce up to 81.0% of unnecessary surgeries.

According to a review and meta-analysis study published by Vargas-Salas and colleagues in 2018 (17), a sensitivity of 92% and specificity of 80% appear to be ideal for an indeterminate thyroid nodule molecular classifier test to have an appropriate clinical performance within a wide cancer prevalence range (20%–40%), reaching at least an NPV of 94% and a PPV of 60% (17). According to these thresholds, only the mir-THYpe, ThyroSeq v3, and ThyroidPrint tests currently present or exceed, within the same test, the proposed thresholds (see Table 4) and can be considered “rule-out” and “rule-in” tests. Although the NPV is the preferred statistical parameter that clinicians consider when evaluating a thyroid molecular classifier test to reduce unnecessary surgeries, reinforcing that this parameter (as the PPV) is highly influenced by the cancer prevalence of the study is highly important (Fig. 1). The NPV prediction of the mir-THYpe test across all of the different cancer prevalence rates from other compared studies (Table 5) shows that the mir-THYpe is predicted to have an NPV lower than 94% (93.1%) only at a 52.6% cancer prevalence (“ThyroSeq v3” study).

It is important to caution that all the data used here to compare the latest version of each molecular classifier test were published very recently (Rosetta GX Reveal and ThyroidPrint in 2017; Afirma GSC and ThyroSeq v3 in 2018; except ThyraMIR/ThyGenX published in 2015), therefore limited information is published about the prospective clinical performance of the latest version of each test, including the mir-THYpe here described.

In summary, the reported data suggest that the mir-THYpe test could be considered for use in clinical practice as a complementary tool to support a more informed clinical decision for patients with indeterminate thyroid nodules and potentially contribute to a reduction in the rates of unnecessary thyroid surgeries and unnecessary expenses by the healthcare system. Moreover, our data show that the mir-THYpe can be considered both a malignancy “rule-in” and “rule-out” test, and can be readily performed using already available cytology slides at a significantly lower cost. Yet, prospective studies assessing the mir-THYpe performance in the clinical setting should be carried out to validate its routine use in clinical practice and its cost-effectiveness.

## Supplementary Material

Supplemental data
