# Supplementary material for: Molecular Classification of Thyroid Nodules with Indeterminate Cytology: Development and Validation of a Highly Sensitive and Specific New miRNA-Based Classifier Test Using Fine-Needle Aspiration Smear Slides
Source: Thyroid. 2018 Dec 14;28(12):1618–26. doi: 10.1089/thy.2018.0254 (PMC6308280; doi:10.1089/thy.2018.0254)
Supplement: Supplemental data [file Supp_Data.pdf]

# Supplementary Data

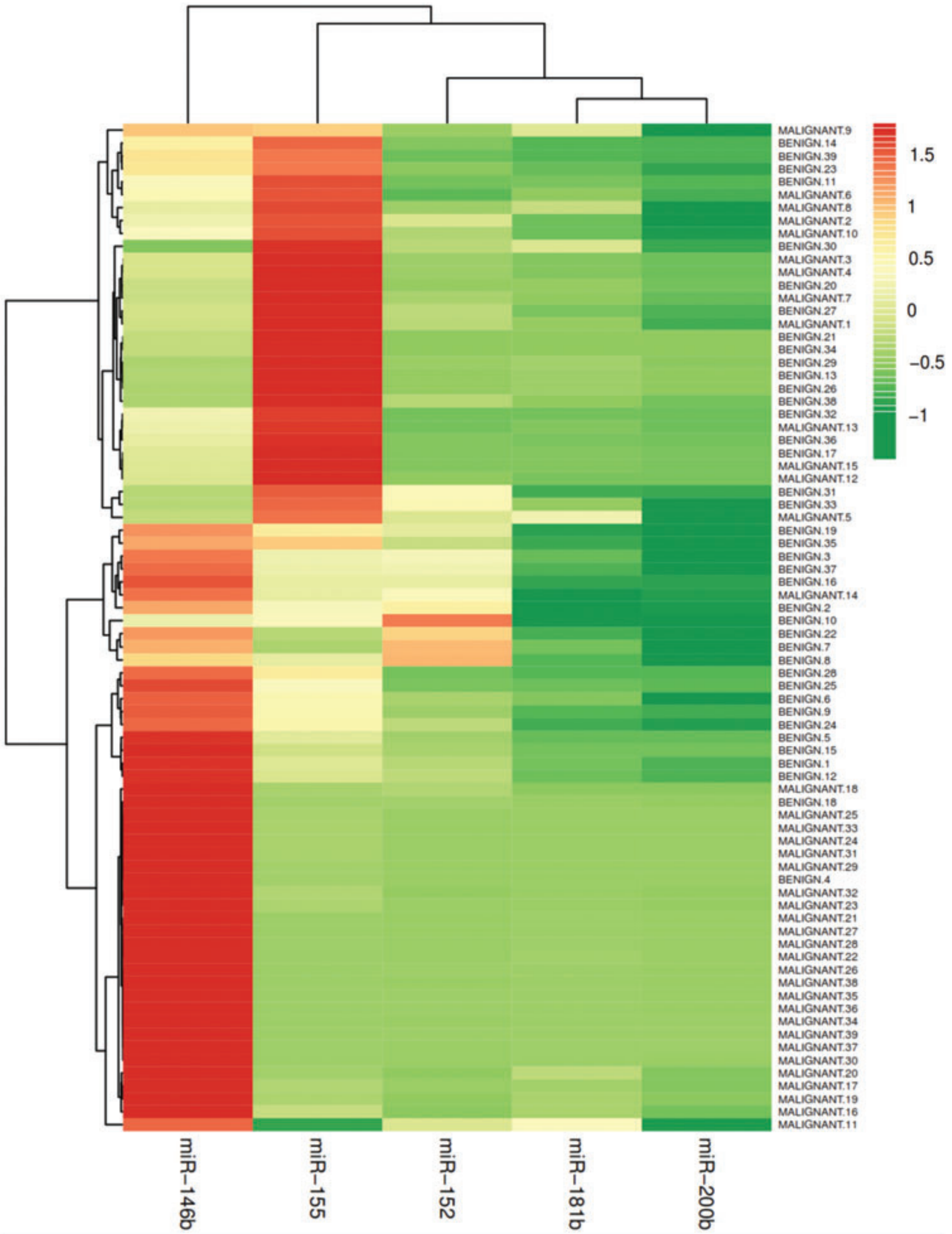


**SUPPLEMENTARY FIG. S1.** Unsupervised hierarchical clustering analysis of normalized expression data from the five discriminator miRNAs used on the mir-THYpe algorithm. The expression levels of each miRNAs as determined by RT-qPCR are color-coded from low expression (green) to high expression (red). Each row is one of the 78 samples from the training set. The label on the right shows the nodule classification by the postsurgical histopathology.The heatmap was designed using the ClastVis webtool (S1).

## References

S1. Metsalu, T and Vilo, J 2015 Clustvis: a web tool for visualizing clustering of multivariate data using Principal Component Analysis and heatmap. Nucleic Acids Res **43:**W566–W570.

Supplementary Table S1. The First Exploratory List of 96 miRNA Candidates

| *No.* | *Assay ID (Thermo Fisher ID)* | *miRNA name* | *Mature miRNA sequence* |
|---|---|---|---|
| 1 | 468 | hsa-miR-146a | UGAGAACUGAAUUCCAUGGGUU |
| 2 | 1097 | hsa-miR-146b | UGAGAACUGAAUUCCAUAGGCU |
| 3 | 397 | hsa-miR-21 | UAGCUUAUCAGACUGAUGUUGA |
| 4 | 2276 | hsa-miR-222 | AGCUACAUCUGGCUACUGGGU |
| 5 | 524 | hsa-miR-221 | AGCUACAUUGUCUGCUGGGUUUC |
| 6 | 382 | hsa-let-7f | UGAGGUAGUAGAUUGUAUAGUU |
| 7 | 2282 | hsa-let-7g | UGAGGUAGUAGUUUGUACAGUU |
| 8 | 416 | hsa-miR-30a-3p | CUUUCAGUCGGAUGUUUGCAGC |
| 9 | 417 | hsa-miR-30a-5p | UGUAAACAUCCUCGACUGGAAG |
| 10 | 2110 | hsa-miR-30c-2* | CUGGGAGAAGGCUGUUUACUCU |
| 11 | 422 | hsa-miR-30e-3p | CUUUCAGUCGGAUGUUUACAGC |
| 12 | 494 | hsa-miR-195 | UAGCAGCACAGAAAUAUUGGC |
| 13 | 502 | hsa-miR-200a | UAACACUGUCUGGUAACGAUGU |
| 14 | 2251 | hsa-miR-200b | UAAUACUGCCUGGUAAUGAUGA |
| 15 | 505 | hsa-miR-200c | UAAUACUGCCGGGUAAUGAUGG |
| 16 | 480 | hsa-miR-181a | AACAUUCAACGCUGUCGGUGAGU |
| 17 | 1098 | hsa-miR-181b | AACAUUCAUUGCUGUCGGUGGG |
| 18 | 2279 | hsa-miR-31 | AGGCAAGAUGCUGGCAUAGCU |
| 19 | 2199 | hsa-miR-125a-3p | ACAGGUGAGGUUCUUGGGAGCC |
| 20 | 449 | hsa-miR-125b | UCCCUGAGACCCUAACUUGUGA |
| 21 | 2246 | hsa-miR-133a | UUUGGUCCCCUUCAACCAGCUG |
| 22 | 2304 | hsa-miR-199a-3p | ACAGUAGUCUGCACAUUGGUUA |
| 23 | 2623 | hsa-miR-155 | UUAAUGCUAAUCGUGAUAGGGGU |
| 24 | 1141 | mmu-miR-451 | AAACCGUUACCAUUACUGAGUU |
| 25 | 391 | hsa-miR-16 | UAGCAGCACGUAAAUAUUGGCG |
| 26 | 564 | hsa-miR-375 | UUUGUUCGUUCGGCUCGCGUGA |
| 27 | 553 | hsa-miR-346 | UGUCUGCCCGCAUGCCUGCCUCU |
| 28 | 2642 | hsa-miR-151-5P | UCGAGGAGCUCACAGUCUAGU |
| 29 | 1094 | RNU44 | CCTGGATGATGATAGCAAATGCTGACTGAA CATGAAGGTCTTAATTAGCTCTAACTGACT |
| 30 | 2269 | hsa-miR-183 | UAUGGCACUGGUAGAAUUCACU |
| 31 | 426 | hsa-miR-34a | UGGCAGUGUCUUAGCUGGUUGU |
| 32 | 475 | hsa-miR-152 | UCAGUGCAUGACAGAACUUGG |
| 33 | 508 | hsa-miR-204 | UUCCCUUUGUCAUCCUAUGCCU |
| 34 | 389 | hsa-miR-15a | UAGCAGCACAUAAUGGUUUGUG |
| 35 | 2255 | hsa-miR-149 | UCUGGCUCCGUGUCUUCACUCCC |
| 36 | 2308 | hsa-miR-17 | CAAAGUGCUUACAGUGCAGGUAG |
| 37 | 2112 | hsa-miR-29a | UAGCACCAUCUGAAAUCGGUUA |
| 38 | 2228 | hsa-miR-126 | UCGUACCGUGAGUAAUAAUGCG |
| 39 | 2284 | hsa-miR-138 | AGCUGGUGUUGUGAAUCAGGCCG |
| 40 | 387 | hsa-miR-10a | UACCCUGUAGAUCCGAAUUUGUG |
| 41 | 405 | hsa-miR-26a | UUCAAGUAAUCCAGGAUAGGCU |
| 42 | 407 | hsa-miR-26b | UUCAAGUAAUUCAGGAUAGGU |
| 43 | 400 | hsa-miR-23b | AUCACAUUGCCAGGGAUUACC |
| 44 | 435 | hsa-miR-99a | AACCCGUAGAUCCGAUCUUGUG |
| 45 | 2422 | hsa-miR-18a | UAAGGUGCAUCUAGUGCAGAUAG |
| 46 | 2217 | hsa-miR-18b | UAAGGUGCAUCUAGUGCAGUUAG |
| 47 | 580 | hsa-miR-20a | UAAAGUGCUUAUAGUGCAGGUAG |
| 48 | 1014 | hsa-miR-20b | CAAAGUGCUCAUAGUGCAGGUAG |
| 49 | 442 | hsa-miR-106b | UAAAGUGCUGACAGUGCAGAU |
| 50 | 456 | hsa-miR-130b | CAGUGCAAUGAUGAAAGGGCAU |
| 51 | 2169 | hsa-miR-106a | AAAAGUGCUUACAGUGCAGGUAG |
| 52 | 1020 | hsa-miR-365 | UAAUGCCCCUAAAAAUCCUUAU |
| 53 | 1516 | hsa-miR-425-5p | AAUGACACGAUCACUCCCGUUGA |
| 54 | 507 | hsa-miR-203 | GUGAAAUGUUUAGGACCACUAG |
| 55 | 473 | hsa-miR-150 | UCUCCCAACCCUUGUACCAGUG |
| 56 | 377 | hsa-let-7a | UGAGGUAGUAGGUUGUAUAGUU |
| 57 | 2619 | hsa-let-7b | UGAGGUAGUAGGUUGUGUGGUU |
| 58 | 2406 | hsa-let-7e | UGAGGUAGGAGGUUGUAUAGUU |
| 59 | 1973 | U6 snRNA | - |
| 60 | 2198 | hsa-miR-125a-5p | UCCCUGAGACCCUUUAACCUGUGA |
| 61 | 497 | hsa-miR-197 | UUCACCACCUUCUCCACCCAGC |

(*continued*)

Supplementary Table S1. (Continued)

| *No.* | *Assay ID (Thermo Fisher ID)* | *miRNA name* | *Mature miRNA sequence* |
|---|---|---|---|
| 62 | 1006 | RNU48 | GATGACCCCAGGTAACTCTGAGTGTGTCG CTGATGCCATCACCGCAGCGCTCTGACC |
| 63 | 439 | hsa-miR-103 | AGCAGCAUUGUACAGGGCUAUGA |
| 64 | 2299 | hsa-miR-191 | CAACGGAAUCCCAAAAGCAGCUG |
| 65 | 2278 | hsa-miR-145 | GUCCAGUUUUCCCAGGAAUCCCU |
| 66 | 2245 | hsa-miR-122 | UGGAGUGUGACAAUGGUGUUUG |
| 67 | 500 | hsa-miR-199b | CCCAGUGUUUAGACUAUCUGUUC |
| 68 | 2253 | hsa-miR-101 | UACAGUACUGUGAUAACUGAA |
| 69 | 1586 | hsa-miR-613 | AGGAAUGUUCCUUCUUUGCC |
| 70 | 2296 | hsa-miR-885-5p | UCCAUUACACUACCCUGCCUCU |
| 71 | 268 | dme-miR-7 | UGGAAGACUAGUGAUUUUGUUGU |
| 72 | 2314 | hsa-miR-7-2* | CAACAAAUCCCAGUCUACCUAA |
| 73 | 1129 | mmu-miR-137 | UUAUUGCUUAAGAAUACGCGUAG |
| 74 | 604 | hsa-miR-424 | CAGCAGCAAUUCAUGUUUUGAA |
| 75 | 1193 | mmu-miR-187 | UCGUGUCUUGUGUUGCAGCCGG |
| 76 | 428 | hsa-miR-34c | AGGCAGUGUAGUUAGCUGAUUGC |
| 77 | 583 | hsa-miR-9 | UCUUUGGUUAUCUAGCUGUAUGA |
| 78 | 1571 | hsa-miR-608 | AGGGGUGGUGUUGGGACAGCUCCGU |
| 79 | 2676 | hsa-miR-144 | UACAGUAUAGAUGAUGUACU |
| 80 | 1608 | hsa-miR-449b | AGGCAGUGUAUUGUUAGCUGGC |
| 81 | 2222 | hsa-miR-1 | UGGAAUGUAAAGAAGUAUGUAU |
| 82 | 2290 | hsa-miR-208b | AUAAGACGAACAAAAGGUUUGU |
| 83 | 511 | hsa-miR-208 | AUAAGACGAGCAAAAAGCUUGU |
| 84 | 2176 | hsa-miR-933 | UGUGCGCAGGGAGACCUCUCCC |
| 85 | 1048 | hsa-miR-503 | UAGCAGCGGGAACAGUUCUGCAG |
| 86 | 509 | hsa-miR-205 | UCCUUCAUUCCACCGGAGUCUG |
| 87 | 592 | hsa-miR-136 | ACUCCAUUUGUUUUGAUGAUGGA |
| 88 | 1116 | hsa-miR-520b | AAAGUGCUUCCUUUUAGAGGG |
| 89 | 1593 | hsa-miR-618 | AAACUCUACUUGUCCUUCUGAGU |
| 90 | 489 | hsa-miR-190 | UGAUAUGUUUGAUAUAUUAGGU |
| 91 | 2776 | hsa-miR-1179 | AAGCAUUCUUUCAUUGGUUGG |
| 92 | 1604 | hsa-miR-651 | UUUAGGAUAAGCUUGACUUUUG |
| 93 | 243597_mat | hsa-miR-3151 | GGUGGGGCAAUGGGAUCAGGU |
| 94 | 1592 | hsa-miR-642 | GUCCCUCUCCAAAUGUGUCUUG |
| 95 | 533 | hsa-miR-302c | UAAGUGCUUCCAUGUUUCAGUGG |
| 96 | 2100 | hsa-miR-136* | CAUCAUCGUCUCAAAUGAGUCU |

Supplementary Table S2. Histological Subtypes for Each Bethesda Class

| | *Training Set (Postsurgical Tissue)* | | | | | | | *Validation Set (FNA Smear Slides)* | | | | | | |
|---|---|---|---|---|---|---|---|---|---|---|---|---|---|---|
| *Histological Subtypes* | *AUS/FLUS (III)* | | *FN/SFN (IV)* | | *SUSP (V)* | | *Total* | *AUS/FLUS (III)* | | *FN/SFN (IV)* | | *SUSP (V)* | | *Total* |
| **Benign** | **14** | **(36%)** | **18** | **(46%)** | **7** | **(18%)** | **39** | **17** | **(29%)** | **39** | **(67%)** | **2** | **(3%)** | **58** |
| Hüthle cell adenoma | 1 | (25%) | 3 | (75%) | 0 | (0%) | 4 | 1 | (13%) | 7 | (88%) | 0 | (0%) | 8 |
| Follicular adenoma | 3 | (75%) | 1 | (25%) | 0 | (0%) | 4 | 2 | (14%) | 12 | (86%) | 0 | (0%) | 14 |
| Colloid goiter | 5 | (33%) | 6 | (40%) | 4 | (27%) | 15 | 4 | (33%) | 6 | (50%) | 2 | (17%) | 12 |
| Adenomatous goiter/ follicular hyperplasia | 1 | (13%) | 6 | (75%) | 1 | (13%) | 8 | 8 | (57%) | 6 | (43%) | 0 | (0%) | 14 |
| Hashimoto's thyroiditis | 1 | (100%) | 0 | (0%) | 0 | (0%) | 1 | 0 | (0%) | 1 | (100%) | 0 | (0%) | 1 |
| Lymphocytic thyroiditis | 2 | (40%) | 1 | (20%) | 2 | (40%) | 5 | 2 | (22%) | 7 | (78%) | 0 | (0%) | 9 |
| Chronic thyroiditis | 1 | (50%) | 1 | (50%) | 0 | (0%) | 2 | 0 | (0%) | 0 | (0%) | 0 | (0%) | 0 |
| **Malignant** | **8** | **(21%)** | **14** | **(36%)** | **17** | **(44%)** | **39** | **1** | **(3%)** | **6** | **(16%)** | **30** | **(81%)** | **37** |
| Papillary thyroid carcinoma, usual type | 3 | (19%) | 2 | (13%) | 11 | (69%) | 16 | 1 | (5%) | 0 | (0%) | 18 | (95%) | 19 |
| Papillary thyroid carcinoma, follicular variant | 3 | (21%) | 6 | (43%) | 5 | (36%) | 14 | 0 | (0%) | 1 | (8%) | 11 | (92%) | 12 |
| Follicular thyroid carcinoma, widely invasive | 0 | (0%) | 2 | (100%) | 0 | (0%) | 2 | 0 | (0%) | 0 | (0%) | 0 | (0%) | 0 |
| Follicular thyroid carcinoma, microinvasive | 0 | (0%) | 0 | (0%) | 0 | (0%) | 0 | 0 | (0%) | 1 | (100%) | 0 | (0%) | 1 |
| Follicular thyroid carcinoma, oncocytic variant | 0 | (0%) | 4 | (80%) | 1 | (20%) | 5 | 0 | (0%) | 1 | (100%) | 0 | (0%) | 1 |
| NIFTP | 0 | (0%) | 0 | (0%) | 0 | (0%) | 0 | 0 | (0%) | 2 | (67%) | 1 | (33%) | 3 |
| Medullary thyroid carcinoma | 2 | (100%) | 0 | (0%) | 0 | (0%) | 2 | 0 | (0%) | 0 | (0%) | 0 | (0%) | 0 |
| Insular thyroid carcinoma | 0 | (0%) | 0 | (0%) | 0 | (0%) | 0 | 0 | (0%) | 1 | (100%) | 0 | (0%) | 1 |
| **Total** | **22** | **(28%)** | **32** | **(41%)** | **24** | **(31%)** | **78** | **18** | **(19%)** | **45** | **(47%)** | **32** | **(34%)** | **95** |

AUS/FLUS, atypia of undetermined significance/follicular lesion of undetermined significance; FN/SFN, follicular or oncocytic (Hürthle cell) neoplasm/suspicious for a follicular or oncocytic (Hürthle cell) neoplasm; NIFTP, noninvasive follicular thyroid neoplasm with papillary-like nuclear features; SUSP, suspicious for malignancy.
